# Inherent X-Linked Genetic Variability and Cellular Mosaicism Unique to Females Contribute to Sex-Related Differences in the Innate Immune Response

**DOI:** 10.3389/fimmu.2017.01455

**Published:** 2017-11-13

**Authors:** Zoltan Spolarics, Geber Peña, Yong Qin, Robert J. Donnelly, David H. Livingston

**Affiliations:** ^1^Department of Surgery, Rutgers-New Jersey Medical School, Newark, NJ, United States; ^2^Department of Pathology and Laboratory Medicine, Rutgers-New Jersey Medical School, Newark, NJ, United States

**Keywords:** sexual dimorphism, X chromosome inactivation, cellular mosaicism, X chromosome skewing, infection, injury, sepsis

## Abstract

Females have a longer lifespan and better general health than males. Considerable number of studies also demonstrated that, after trauma and sepsis, females present better outcomes as compared to males indicating sex-related differences in the innate immune response. The current notion is that differences in the immuno-modulatory effects of sex hormones are the underlying causative mechanism. However, the field remains controversial and the exclusive role of sex hormones has been challenged. Here, we propose that polymorphic X-linked immune competent genes, which are abundant in the population are important players in sex-based immuno-modulation and play a key role in causing sex-related outcome differences following trauma or sepsis. We describe the differences in X chromosome (ChrX) regulation between males and females and its consequences in the context of common X-linked polymorphisms at the individual as well as population level. We also discuss the potential pathophysiological and immune-modulatory aspects of ChrX cellular mosaicism, which is unique to females and how this may contribute to sex-biased immune-modulation. The potential confounding effects of ChrX skewing of cell progenitors at the bone marrow is also presented together with aspects of acute trauma-induced *de novo* ChrX skewing at the periphery. In support of the hypothesis, novel observations indicating ChrX skewing in a female trauma cohort as well as case studies depicting the temporal relationship between trauma-induced cellular skewing and the clinical course are also described. Finally, we list and discuss a selected set of polymorphic X-linked genes, which are frequent in the population and have key regulatory or metabolic functions in the innate immune response and, therefore, are primary candidates for mediating sex-biased immune responses. We conclude that sex-related differences in a variety of disease processes including the innate inflammatory response to injury and infection may be related to the abundance of X-linked polymorphic immune-competent genes, differences in ChrX regulation, and inheritance patterns between the sexes and the presence of X-linked cellular mosaicism, which is unique to females.

## Introduction

Females have better general health and a longer lifespan than males (1, 2). Sex-related disparities in the immune response show a mixed pattern. Whereas autoimmune diseases are predominant in females (3–6), a large number of studies demonstrated that following trauma and sepsis females present improved clinical course and better outcomes as compared to males (7–22). However, the field remains controversial as some investigations showed no female benefit or a few studies even suggested a female disadvantage following infection (23–25). These controversies may be related to the fact that most human studies were designed to simply compare males versus females or after stratification by sex hormone status without considering genetic contributions (26–28). Animal studies using genetically homogenous inbred strains are more convincing in showing a female benefit but the aspect of genetic polymorphism as it exists in humans is lacking in these models.

Whereas ample evidence exists to support the important immuno-modulatory role of sex hormones (7–13, 26–32), sex-based outcome differences associated with the innate immune response are unlikely to be solely dependent on their effects. For example, observations on young prepubertal children indicated that boys have higher mortality rate than girls after burns (33). Infection-induced mortality rate was also higher in newborn boys than girls (34, 35). Male newborns displayed more frequent viral and bacterial respiratory infections and were more prone to sepsis and meningitis than girls (35). Additionally, sex differences in disease processes including sepsis were shown to manifest between postmenopausal women and older men (36, 37). Even animal models using castrated or ovariectomized animals or sex hormone replacements found that sex-related differences were only partially diminished between males and females (38–40). Finally, studies testing the beneficial effects of sex hormone treatment after injury or sepsis employed pharmacological doses of estrogen. Therefore steroid-like effects or secondary changes in the ACTH axis may have contributed to the specific hormonal effects (41–46). These observations indicate that factors other than sex hormones likely contribute to sex-dimorphic outcomes (20, 21, 35, 47, 48). Whereas studies have started to address the role of X chromosome (ChrX) in autoimmune diseases (3–6), the role of sex chromosomes in contributing to sex-related immunomodulation during the innate immune response has not been thoroughly examined.

We previously proposed that common non-pathological X-linked genetic polymorphisms may be important in modulating sex-based differences in innate immunity and neglecting the genetic aspect may be part of the reasons of current controversies in this field (16, 48). The proposed ChrX hypothesis has been tested and confirmed in animal proof-of-concept studies (49–51) and recently published human investigations also support the concept (52). The aim of the current article is to present the hypothesis in a new light and in the context of recent clinical observations. We present how the inherently polymorphic ChrX and uniquely female ChrX mosaicism may contribute to immuno-modulation at the individual subject as well as human population level. Furthermore, we identify and discuss selected polymorphic X-linked candidate genes, which we believe are likely players in sex-based immunomodulation *via* cellular mosaicism and sex-linked inheritance patterns. New experimental findings will also be shown. Aspects of autoimmune diseases, sex hormone effects, and the potential contribution of Y chromosome genes will be discussed only to the extent that it is related to the proposed concept.

## ChrX-Mosaicism and Cellular Variability

**Figure 1:**
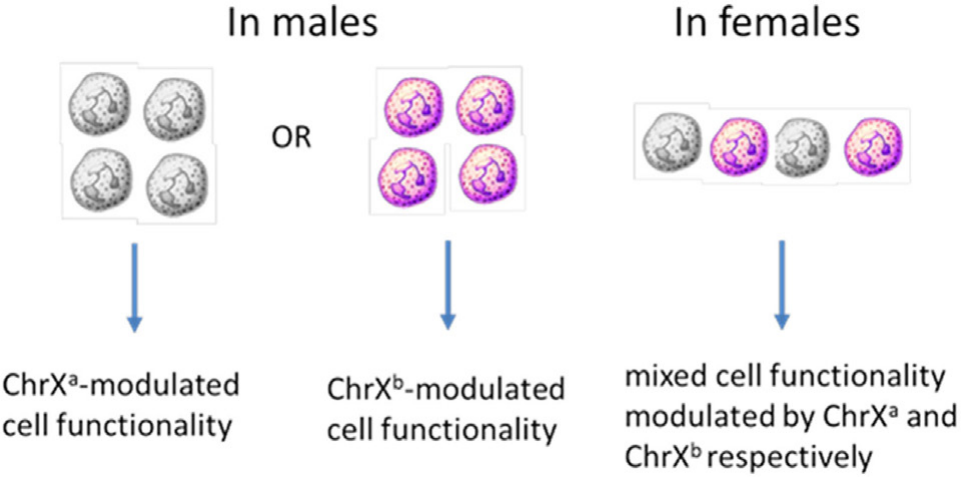
ChrX-linked cellular mosaicism for the expression of variant proteins results in phenotype diversity in females but increased functional polarity in males.

Females inherit two naturally polymorphic parental X chromosomes (ChrXs) whereas males inherit a single ChrX passed on from the mother. Therefore, females carry polymorphic X-linked alleles from both parents while males carry only the maternal variants (53, 54). To compensate for the potential double-dosed gene expression in females as compared to males, cells undergo random ChrX inactivation during early female embryonic development. The process involves methylation of one of the ChrXs rendering it inactive for gene expression (55–57). Although the cells retain and copy the inactive DNA during cell divisions, the expression-block is retained for the entire lifespan of the cell. These epigenetic changes and the random selection of ChrX inactivation within a cell result in females showing cellular mosaicism for the expression of X-linked polymorphic proteins or differences in gene regulation due to allelic variations between mosaic cells. The ratio of circulating blood cells with the active respective parental ChrXs, on average, approximates to one-to-one in younger healthy individuals (56–58). In blood, mosaic cell populations are dispersed homogenously, however, because organs start developing from cellular islands during embryogenesis, the distribution of X-linked mosaicism is patch-like and inhomogeneous in solid organs and tissues (59). The likely consequence of X-linked mosaicism is that the innate genetic polymorphisms of the ChrXs will result in distinct variations in mosaic cellular subsets resulting in phenotypes with different regulatory potentials or functional responsiveness within a female subject (48). This is in contrast with males who lack X-linked cell mosaicism and in whom polymorphisms from the maternal ChrX are the only drivers of X-linked cellular variability (Figure 1). Based on these considerations, we propose that increased cellular variability through ChrX mosaicism in otherwise healthy females may be an advantageous condition during the innate immune response by providing improved functional flexibility to dynamically changing pathophysiological conditions. These differences in cellular phenotypes may be a contributing factor to sex-based outcome differences and immune-modulation. The concept of improved cellular adaptability in females is supported by the fact that ChrX skewing is frequently observed in healthy females or in severe X-linked genetic defects and it is defined as increased numbers of one of the mosaic subpopulations relative to the other (60–62).

**Figure 2:**
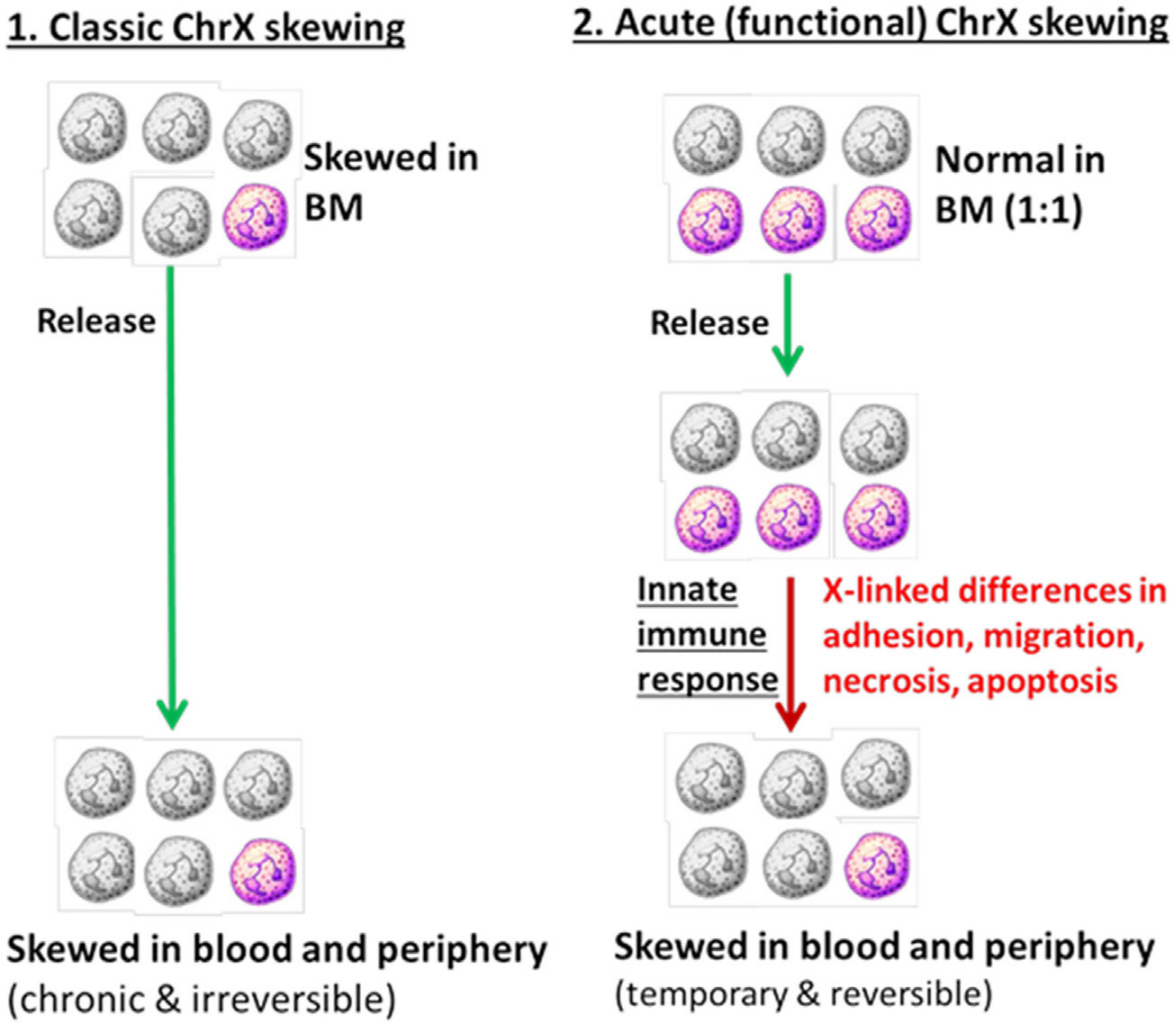
Cellular skewing driven by primary changes in bone marrow progenitors (Part 1) versus selection of mosaic subpopulations at the periphery driven by X-linked allelic variants (Part 2).

During inflammation, *de novo*, acute and functional ChrX cellular skewing may occur resulting from different degrees of cell trafficking, recruitment to injury or infection sites, or dissimilar rates in necrosis, apoptosis, and cell proliferation driven by X-linked allelic variations (Figure 2). This *de novo* X-linked cellular skewing is expected to be temporary and reversible. When the inflammatory response dissipates and infection or injury resolves, the original cell ratio is expected to become rebalanced. The exception is when the inflammatory response results in irreversibly skewed cell ratios in bone marrow progenitors, which is an unlikely scenario. Our previous observations from mice studies using X-linked knockout and mosaic models for CYBB (gp91phox) and interleukin-1 receptor-associated kinase 1 (IRAK1) support this hypothesis (49–51). These studies showed similar mosaic cell ratios between BM and blood in healthy animals. However, endotoxemia or sepsis resulted in ChrX skewing in circulating blood and immune-competent organs. These investigations also revealed that animals with ChrX-linked mosaicism showed improved outcome as compared to WT or deficient animals but the mechanisms of protection were different in the gp91phox- and IRAK1-deficient models (49–51).

Observations from these animal studies also suggested that the presence of different mosaic cell populations may provide the conditions for intercellular communication between mosaic cells. An interplay among mosaic cellular variants in females could “buffer” the inflammatory response by downregulating hyper-active cell populations during excessive inflammation or in contrast, may compensate for immuno-paralysis, thereby improving the clinical course. Evidently, this potentially beneficial “buffering” mechanism is lacking in single-ChrX males.

However, it needs mentioning that ChrX skewing may also be disadvantageous in some cases. For example, gross and spontaneous ChrX skewing of BM progenitors in healthy females may precondition immune cells for a “biased” cell response to injury or infection driven by polymorphisms of one of the ChrXs. Moreover, extreme ChrX skewing in a female manifests a male-like phenotype because cells expressing one of the two ChrXs dominate in the circulation. This possibility is realistic as spontaneous ChrX cellular skewing in the bone marrow and blood is common in otherwise healthy females especially at older age (63–65).

## Impacts of Common X-Linked Polymorphisms at the Population Level

The potential effects of X-linked polymorphisms in the context of sex-based outcome differences are expected to be significant due to several factors (66). First, because of the X-linked inheritance pattern, it is evident that all clinically important X-linked polymorphisms have a greater impact on the male than female population in contrast with polymorphic alleles linked to the autosomal chromosome, which show no sex bias (48, 53). For example, a 20% allele frequency of an X-linked variant allele in a randomly mating population or a population sample showing Hardy–Weinberg equilibrium will result in genotype frequencies in females as 4% affected (homozygous), 32% mosaic, and 64% normal. In males of the same population, the genotype distribution will be 20% affected (hemizygous) and 80% normal, which indicates that males are affected five times more than females in this example. In addition, a large segment of the female population presents cellular mosaicism for expressing the WT and risk allele, respectively (48). Females who have mosaicism for the expression of the WT and risk alleles are likely to present their own phenotypes, which are different from females carrying cells homozygous either for the variant or WT alleles as demonstrated in our mice studies (49–51). These facts indicate that part of the female health advantages over males could be related to, or at least impacted by, sex-biased distributions of common X-linked polymorphic alleles together with the presence of uniquely female X-linked cellular mosaicism (48).

**Figure 3:**
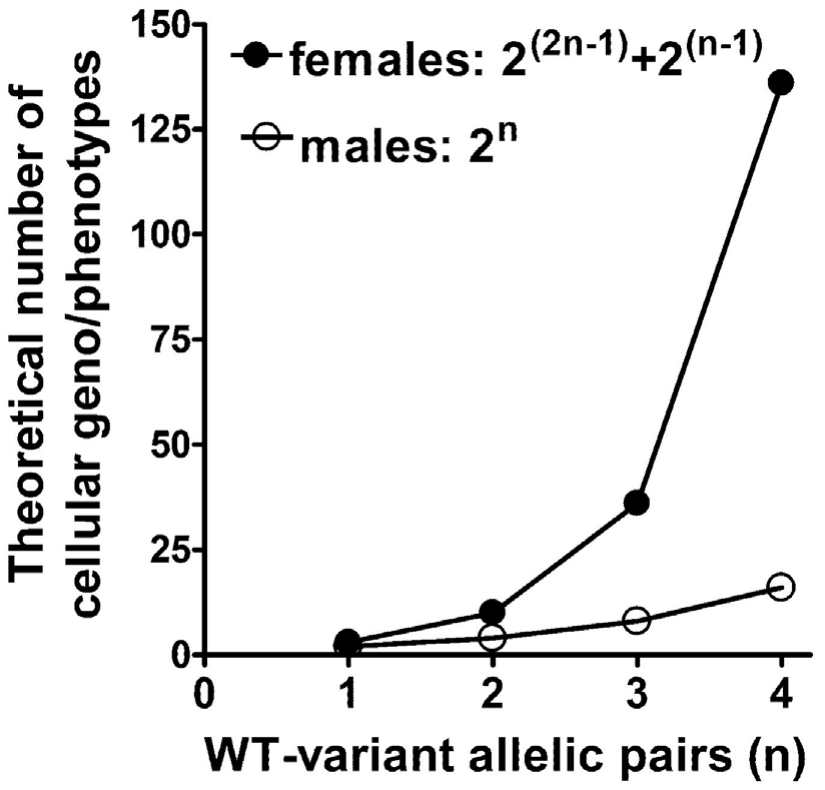
Possible number of different cellular phenotypes in female ChrX mosaicisms versus single-ChrX carrying males. The number of cellular phenotypes are shown as the function of the number of functionally interrelated polymorphic gene variant pairs which reside in different recombination regions of the ChrX.

Second, X-linked cellular variability becomes even more complex after considering the possibilities produced by gametal ChrX recombination in mothers followed by subsequent random ChrX inactivation in female embryos. Sex chromosome recombination is different from that of autosomal chromosomes because recombination between two ChrXs can take place only in females. In males, recombination between the sex chromosomes is limited to the small pseudo-autosomal regions at the tips of the Y and X chromosomes (59). For example, consider two X-linked alleles producing functionally related or interacting protein A and B and their allelic variants a and b in a population. If these two related allelic mutations reside in different recombination blocks, then recombination followed by ChrX inactivation theoretically will result in 10 different mosaic cell pairs with associated phenotypes in females: AB + AB, AB + Ab, AB + aB, AB + ab, Ab + Ab, Ab + ab, Ab + aB, aB + aB, aB + ab, ab + ab. However, in males, the potential genotypic combinations are limited to produce only four cellular phenotypes in the absence of cellular mosaicism (AB, Ab, aB, ab). Assuming more interrelated but independently recombined X-linked variants, the male–female difference in the possible numbers of mosaic phenotypes increases exponentially reaching almost a 100-fold difference at four related alleles (Figure 3). Most of these combinations are not likely to produce distinct phenotypes in females and the X-linked pattern distorts the population frequency of these genotype–phenotype combinations; however, the fact remains unquestionable that the potential for a markedly increased variability to manifest in females as compared to males is there. Furthermore, studies have also indicated that up to 15% of genes may escape permanent silencing on the inactive ChrX (55, 59), which may also add to increased cellular diversity in females compared to males. These facts not only indicate inherent difficulties in predicting phenotype-genotype relationships in the context of sex but also complicate the evaluation of genetic association studies testing X-linked genes (48, 53).

**Figure 4:**
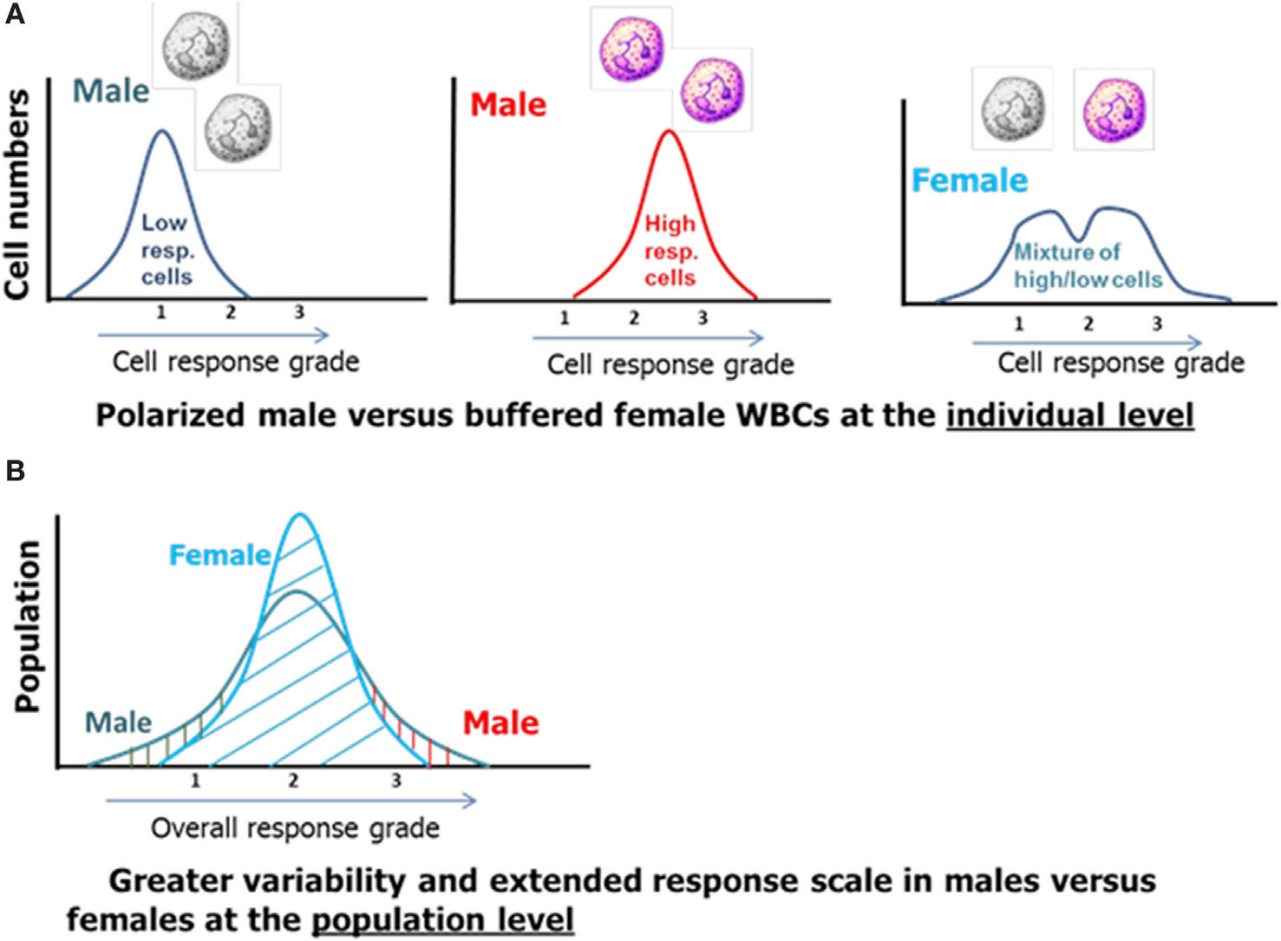
The effect of X-linked cellular mosaicism in females and its absence in males at the individual **(A)** and population level **(B)**.

The facts and considerations presented so far indicate that X-linked polymorphisms including known risk alleles together with other common allelic variations with immune-modulatory potentials are likely contributors to sex-based differences in the innate immune response and post-injury recovery. The unique distribution and different regulation of ChrXs between the sexes present a more polarized cellular machinery in males as compared to females at the individual level (Figure 4, top). The duality of cellular machinery in females should blunt polarity resulting in buffered responses either by simply “diluting” polarized cells or through cell-cell interactions and consequent positive and negative feedback mechanisms (Figure 4A). At the population level, these X-linked differences are expected to result in an increased representation of males at the extreme boundaries of the response distribution curve (Figure 4B).

In summary, in any clinical cohorts of mixed sex, males hemizygous for the risk allele will always be more represented than homozygous females and a large segment of the female subject will show mosaicism for the alleles with their own unique phenotypes. Subjects who show mosaicism for the expression of the variant and WT alleles may also be further subdivided by the presence or absence of spontaneous or pathology-induced ChrX skewing. In those individuals who present ChrX skewing, it also needs to be tested whether the “direction” of ChrX skewing manifests cellular subset ratios moving toward or against variant allele-expressing populations. This is important because without knowing the “direction” of ChrX skewing, it is impossible to conclude whether the mosaic subjects are rendered quasi WT or quasi variant phenotype, thereby being protected or prone to pathology. These facts and reflections indicate that genetic association studies investigating cohorts of mixed sex should consider aspects of X-linked polymorphisms, cellular mosaicism, and the status and direction of chronic or acute ChrX skewing in the analyses. This will help in identifying female subgroups within a cohort with distinct genotype–phenotype relationships and associated pathology and thus could help in resolving continuing controversies in the field.

## Testing the Hypothesis in Humans

As discussed earlier, observations from animal studies using X-linked genetic knockouts combined with X-linked cellular mosaicisms supported the proposed concept at the proof-of-principle level (49–51). However, the proposed hypothesis is based on the notion that naturally occurring X-linked risk alleles in combination with other common polymorphisms with immune-modulatory potentials drive sex-related disparities in the innate immune response in humans. Therefore, only human clinical studies can adequately test these questions. To achieve this goal, we postulate that investigating trauma patients may present advantages over other clinical conditions of infection, which developed secondary to immunocompromised status or chronic diseases. The reason is that, in trauma patients, the pathology is initiated suddenly at the time of injury, which is followed by a temporal cascade of events manifesting a sterile non-specific inflammatory response. Those patients who survive their initial injuries frequently present secondary clinical complications, which may manifest in infection leading to sepsis and organ dysfunction and culminates in late post-injury mortality in some patients. Additionally, a large proportion of trauma victims are relatively young or middle-aged, which lessens analytical difficulties related to comorbidities and age. Thus, the well-defined starting point at the time of injury followed by a cascading pathology with chances for secondary complications in a trauma cohort provides a clinical situation, which could manifest acute and functional ChrX skewing.

Our recently published study conducted on 39 female trauma patients with moderate to severe injuries (52) indicated that at admission, two-third of the patients displayed X-linked mosaic cell ratios between 1 and 3. About a third of the patients presented ratios at the 3–7 range and three patients displayed markedly skewed mosaic cell ratios in the 8–30 range. Testing serial blood samples during the clinical course showed additional changes in cell-ratios ranging between 20 and 900% over initial. Changes in mosaic cell ratios during the injury course correlated with the severity of trauma need for ventilator support and development of pneumonia (52).

**Figure 5:**
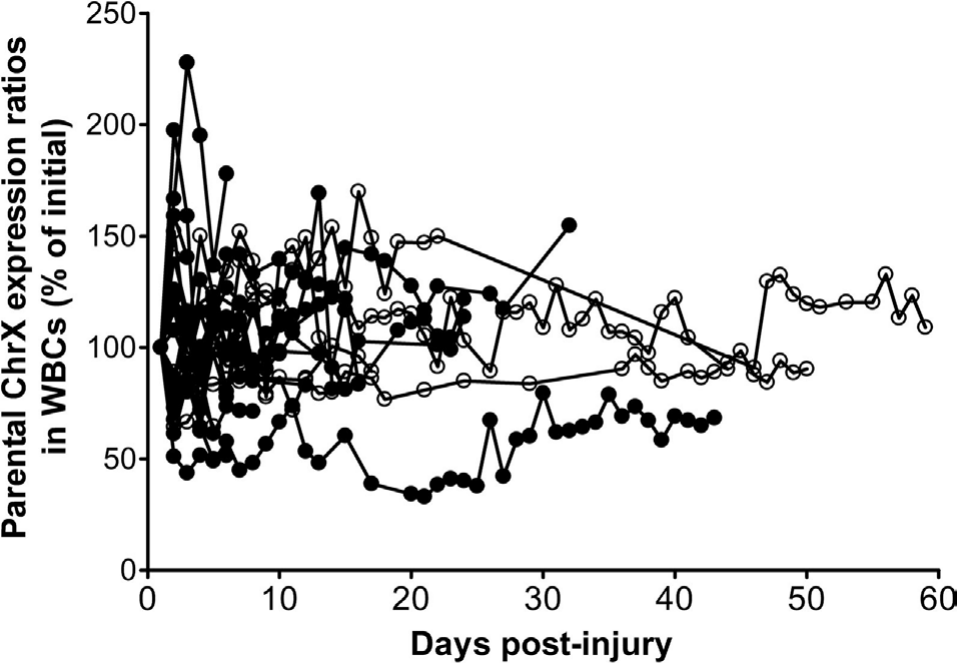
Alterations in ChrX ratios in white blood cell (WBC) during the trauma course. ChrX ratios are expressed as percent of initial measured in the first blood sample drawn at admission. ChrX active/inactive-ratios in circulating WBCs were determined using the variable length polymorphism at the HUMARA locus as we described in detail earlier (52).

We also conducted a follow-up study testing the same question on a subsequent independent trauma cohort with a sample size of 60 (Figure 5). These new observations confirmed previous findings indicating that about a third of the patients presents trauma-induced *de novo* ChrX skewing at a degree greater than 30% over initial during the hospital course (Figure 5). The most marked changes in post-injury ChrX skewing seem to occur within the first 3–6 days after injury. In recovering patients, toward the end of the hospital stay, skewed cell ratios begin to return to initial values of ChrX ratios similar to that found at admission (Figure 5).

To gain more insights into how ChrX skewing may relate to the fine clinical course, we also present actual white blood cell (WBC) numbers expressing respective parental ChrXs from three patients from this cohort to present architypes for typical patterns.

**Figure 6:**
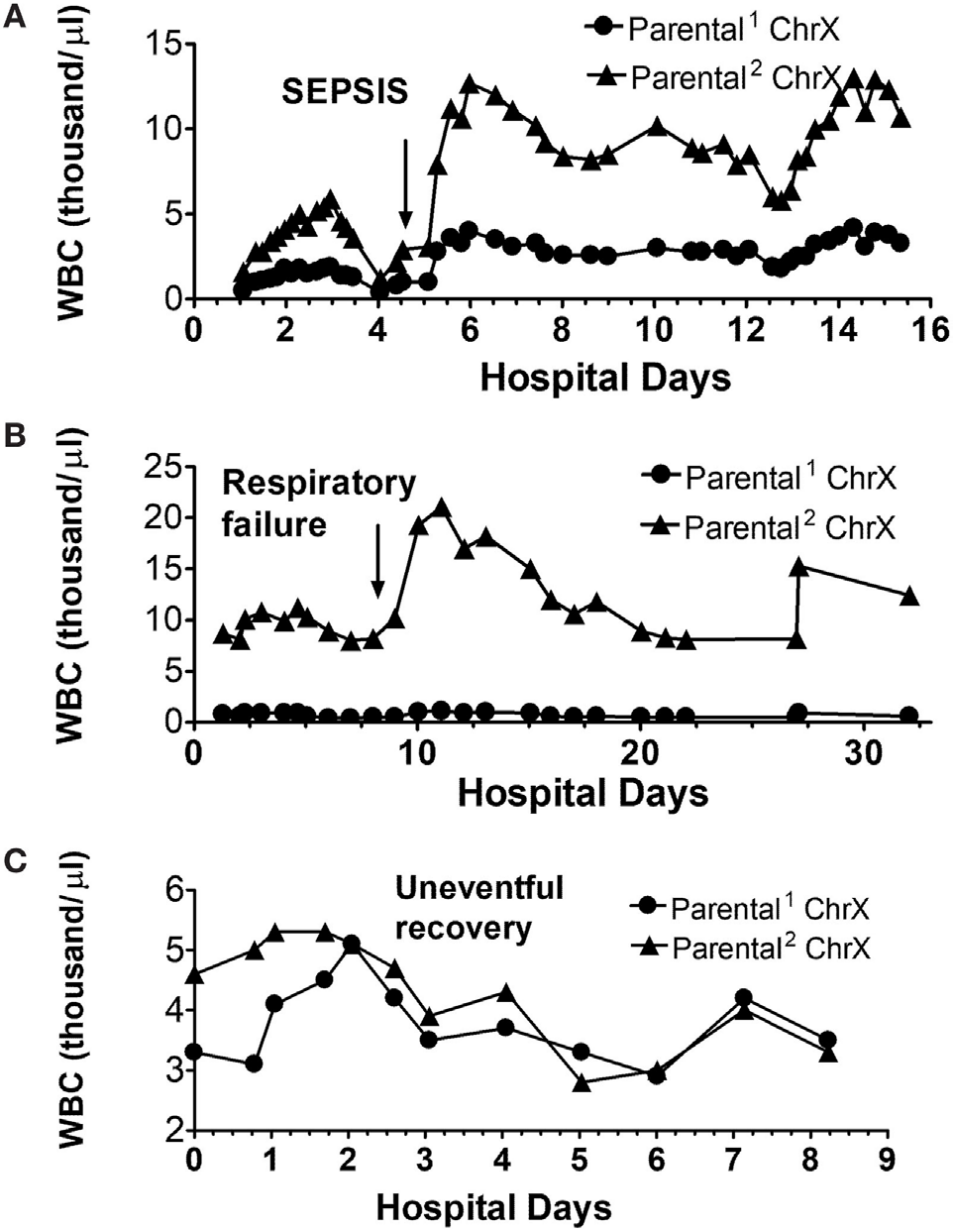
Alterations in circulating white blood cell (WBC) numbers expressing respective parental ChrXs during complicated **(A,B)** and uncomplicated **(C)** clinical courses. ChrX active/inactive-ratios in circulating WBCs from serial samples were determined using the variable length polymorphism at the HUMARA locus as we described in detail earlier (52). From the ChrX ratios combined with the accompanying circulating WBC counts, number of cells expressing respective parental ChrXs were calculated. Arrows indicate the time of onset of sepsis or respiratory failure.

Figure 6A indicates a patient with similar initial WBC numbers expressing respective parental ChrXs measured at admission. There is a transient and reversible increase in X-linked WBC selection peaking at day-3 post injury returning to initial values at day 4. Just after the diagnosis of clinical sepsis on day 4 (arrow), ChrX skewing becomes evident again and lasts for the remaining of the hospital course with only small additional variations.

The second patient (Figure 6B) already showed marked ChrX skewing in WBCs at admission, which was followed by an additional increase of the dominant mosaic subset peaking at day 5 and returning to original levels by day 7. After the diagnosis of respiratory failure, a marked increase in WBC number occurred, but it was restricted to the already dominant mosaic subpopulation of WBCs.

Finally, Figure 6C depicts a patient with a non-complicated “uneventful” hospital course indicating normalization of WBC ChrX skewing by day-2 followed by small variations in WBC numbers without ChrX skewing during the remaining course.

The observed pattern of these case studies is consistent with the proposed hypothesis and supports the notion that X-linked cell selections occur during the inflammatory response to trauma and most likely also manifests after other inflammatory and infectious conditions. Trauma-induced ChrX skewing seems to be in a temporal relationship with the onset and progression of a clinical complication and associated pathophysiological challenges. It however, remains to be tested whether ChrX skewing represents a clinical advantage following trauma as the phenomenon may simply be a manifestation of X-linked functional differences among mosaic subsets without changing clinical outcome. At this point, it cannot be concluded that the outcome advantage of females after trauma is directly associated with increased ChrX skewing. Nevertheless, the potential manifestation of X-linked, variable, buffered, and adaptive cellular responses in females, and the fact that these X-linked mechanism is lacking in males, is likely to benefit most of the females during an innate inflammatory response under a variety of clinical conditions.

## X-Linked Genes of Immuno-Modulation

Past and recent advances in whole genome sequencing, new generation expression profiling, and identifying allelic variations opened revolutionary new possibilities in elucidating genetic components of disease susceptibility and outcome. New allelic variants are identified with increasing speed and the information is readily available from a variety of databases. These robust data sets offer great possibilities for data mining across a variety of genetic backgrounds and pathological conditions. Although the quality of the information in these databases can be inconsistent, for example, some of the genes are studied in great details, others neglected. Therefore, using “educated guesses” in identifying relevant X-linked candidate genes in seeking insights to genotype–phenotype relationships remains a valid and productive approach. In this section, we highlight polymorphic X-linked genes that are widespread in the population and were shown to have immuno-modulatory effects or clinical phenotypes. We propose that allelic polymorphic forms of these genes either alone or in combination are most likely to be responsible or at least contribute to sex-related differences in the immune response and immuno-modulation.

ChrX is medium-sized among chromosomes and carries about 1,200 genes (67). ChrX is relatively rich in highly polymorphic immune competent genes including mediators of the toll-like receptor (TLR)-mediated signaling pathway, TLR7, TLR8, IRAK1, NEMO (IKKγ), NKRF, NKAP, Bruton’s kinase (BTK). Other X-linked proteins with immune competent functions include CD40 ligand (CD40L), FOXP3, IL13R, BMX, IL2RG, and TMP1. In addition, ChrX codes for enzymes involved in redox regulation and apoptosis, NOX1, NOX2, glucose-6-phosphate dehydrogenase (G6PD), XIAP, and AIF (more details in Table 1).

**Table 1:** X-linked genes with major roles in the inflammatory response.

*Cytokine and toll-like receptor (TLR) signaling*

IL1RAPL1 and IL1RAPL2: members of the interleukin-1 receptor family
IL2RG: receptor-γ for IL-2, 4, 7, 9, 15, and 21
IL13RA1 and IL13RA2: decoy receptors for IL-13
CXCR3: chemokine receptor for CXCL 9, 10, and 11
TLR7: toll-like receptor 7
TLR8: toll-like receptor 8
BGN: endogenous ligand for TLR2 and TLR4
Bruton’s kinase: TLR signaling
Interleukin-1 receptor-associated kinase 1: TLR signaling

*NF-kappaB and MAPK signaling*

IKBKG(NEMO): inhibitor of kappa B kinase gamma
NKRF: silencing of IFNB through NF-κB inhibition
NKAP: NF-kappaB activating protein
EDA and EDA2R: ectodysplasin A and its receptor, NF-κB, and JNK regulation
MKP4: inactivation of MAP kinases
CNKSR2: MAPK activation

*Apoptosis, redox balance, and metabolism*

XIAP (BIRC4): direct inhibition of caspase 3 and 7
AIFM1: apoptosis-inducing factor *via* mitochondria
IGBP1: apoptosis inhibition *via* BCR and CD79
Glucose-6-phosphate dehydrogenase: oxidative burst, ROS production, and antioxidant defense (*via* GSH)
NOX1 and NOX2: catalytic units of NADPH oxidases producing superoxide anion and ROS

*Other immuno-modulators*

CD40 ligand: antigen presentation and T cell activation
FOXP3: differentiation of regulatory T cells
MTCP1: T cell proliferation
VSIG4: macrophage phagocytosis and T cell inhibition
BMX: growth and differentiation of hematopoietic cells
TIMP1: wound repair and tissue inflammation
GATA1: differentiation of erythrocytes and megakaryocytes
FGF16: promotes fibroblast in tissue repair and inflammation
GAB3: macrophage differentiation
TSC22D3: anti-inflammatory and immunosuppressive glucocorticoid receptor
PFC: alternative complement pathway regulation
WAS: immune activation, BM function, cytoskeleton (Wiskott–Aldrich protein)
SH2D1A/SAP: B and T cell stimulation
ARHGAP4 and ARHGAP6: early immune cell activation *via* Rho GTPase
DUSP21: anti-inflammatory
ARHGEF9: cell cycle regulation

Interleukin-1 receptor-associated kinase 1 haplotype is one of the primary candidate gene contributing to sex-related outcome differences following trauma and sepsis (68–70). IRAK1 is a member of the intracellular TLR/IL1R/MyD88 adapter complex (71–75). Upon TLR or IL-1β stimuli, IRAK1 becomes phosphorylated and mediates the recruitment and rearrangement of other regulatory proteins, which subsequently leads to IKKγ (NEMO), NFκB, and MAPKs activation resulting in cell activation and cytokine production (71–76). IRAK1 activation is pro-inflammatory (76–78). Animal studies indicate that inhibition or lack of IRAK1 expression is beneficial in sepsis, burn or endotoxemia (79–84).

The human IRAK1 haplotype is composed of seven SNPs five intronic and two exonic. The two exonic polymorphisms results in amino acid (aa) changes at 196 (phe > ser) and 532 (ser > leu). The change at aa 532 seems to be the critical mutation as it persistently increases kinase activity of IRAK1 and augments cell activation (70, 85) in accordance with similar observation from animal studies (79–84).

The clinical impact of IRAK1 variants is well established, as independent studies on septic European as well as Asian cohorts (46, 69, 70) indicated doubled incidence of septic shock, pneumonia, and mortality as compared to WT individuals. In these sepsis studies (69, 70), IRAK1-mosaic females were merged with the WT group, therefore, the effects of mosaicism remained elusive. A later study conducted on trauma patients (68) indicated markedly worsened late post-injury mortality rate of individuals with this haplotype as compared to WT, whereas late mortality rate of “heterozygous” mosaic females was at midway between WT and variant patients. However, the number of homozygous variant and “heterozygous” mosaic female subjects was small in the cohort and aspects or ChrX mosaicism or accompanying ChrX skewing were not examined (68).

The population frequency of the IRAK1 haplotype is 20–40% in whites and Hispanics (86–88). The frequency distribution is reversed in the Asian population, where the variant haplotype may reach 70% prevalence (22, 46, 88, 89). It is important to mention that although most individuals of African descent do not carry the full IRAK1 haplotype, the allelic variation causing the clinically important amino acid change is present at 30–40% frequency. Thus, the X-linked inheritance pattern, high population frequency, and abundance of cellular mosaicism for WT and variant IRAK1 make it likely that the IRAK1 haplotype contributes to sex-biased immuno-modulation across all races and ethnicities.

The variant IRAK1 has also been shown to be associated with increased susceptibility to systemic lupus erythematosus (SLE) (86, 88) an autoimmune disease affecting predominantly females (4, 90, 91). Intriguingly, in the rare cases when SLE develops in males, the clinical course is worsened and mortality is greater than that of observed in females (90, 91). This apparent discrepancy, however, is consistent with the proposed ChrX hypothesis. Namely, cellular mosaicism for X-linked risk alleles including IRAK1 combined with ChrX reactivation (47, 92, 93) could account for the increased susceptibility of females to SLE but, at the same time, ChrX mosaicism may buffer the SLE-associated inflammatory response and alleviate organ dysfunction. In contrast, the lack of ChrX mosaicism in males lessens the chance to the initial onset of SLE but when develops the associated inflammation is more robust due to an “un-buffered” response resulting in more severe organ dysfunction and increased mortality (90, 91). This interconnected relationship of X-linked disease susceptibility, the presence or absence of ChrX mosaicism and associated “immuno-buffering,” or lack of, further signify the complexities of sex-based immuno-modulation.

Among the identified TLRs, TLR7 and TLR8 are X-linked. Clinically important allelic variants have been described impacting susceptibility to tuberculosis, altered macrophage function, allergic and autoimmune conditions including SLE (94, 95) and complications of viral infections (95–101). The population frequency of the minor alleles ranges between 15 and 40% in the European and African population. Interestingly, in the East Asian population, the variant TLR7 and TLR8 alleles are more abundant than the ancestral WT similarly to that observed for IRAK1 population distributions. TLR7 plays an important role in the recognition and subsequent signaling of single-stranded RNA from viruses including influenza, measles, and dengue (102–104). The clinical course of these virus infections was reported to be worse in women than men (24, 25). Polymorphisms of TLR7 and TLR8 have been shown to impact the clinical course of measles (105), but it remains to be tested how ChrX mosaicism for TLR variants impacts outcome after these virus infections.

Nuclear factor kappa B (NFκB) activation is achieved by the phosphorylation of the NFκB-inhibitory proteins (IκB) through the actions of IκB-kinases (IKKα and β). IκB-kinases are in complexes with the X-linked NFκB essential modulator, IKKγ (NEMO) (106–111). Upon a variety of stimuli, including TLR, DAMP, and PAMP activation and subsequent signaling, NEMO becomes activated (112, 113) and interacts with IκB-kinases. Complete lack of NEMO activity is lethal in males, whereas heterozygous mosaic females present an immune-compromised clinical condition (incontinentia pigmenti) (114, 115). The reported SNPs on exons, introns, and flexing gene regions are increasing. Some of these new mutations are associated with complex immune pathologies. Thus, polymorphic forms of NEMO together with allelic variants of other X-linked members of the NFκB regulatory cascade (Table 1) are likely candidates for sex-related immuno modulation.

Bruton’s kinase also participates in TLR-mediated NFκB and p38 MAPK activation affecting TNFα, IL-1β, and IL-12 production as well as iNOS expression and Ca^2+^ release (116, 117). Severe BTK mutations causes X-liked agammaglobulinemia (XLA) in humans and X-linked immune deficiency in mice (Xid) (116, 117). Several polymorphic alleles causing different degree of XLA phenotypes have been reported (118–120).

FOXP3 is a key regulator of T cell activation and CD40L a transmembrane signaling molecule involved in platelet, endothelial, and immune cell activation. FOXP3 is highly polymorphic and clinically relevant allelic variants have been reported to be present in the approximate range of 10–35% in the population and allele frequencies show marked differences among various ethnic groups (121). The variants have been shown to be associated with autoimmune syndromes, allergic conditions, and cancer (122–127).

X-linked G6PD is the rate limiting enzyme of the pentose phosphate shunt and supplies reduced NADPH as the electron donor for superoxide production in phagocytes. Through the actions of peroxidases and glutathione reductase, G6PD also plays a critical role in supporting antioxidant pathways (128). G6PD is highly polymorphic. The allele frequencies of the common African and Mediterranean polymorphic forms that results in an 80–90% decrease in the enzyme activity of red blood cells may reach 10–25% in populations living in or migrated from sub-Saharan Africa or other malaria endemic regions (129, 130). Whereas the common African Mediterranean polymorphisms affect primarily red blood cell functions due to decreased antioxidant capacity, polymorphic forms have been shown to modulate cytokine production (131, 132) and worsen the clinical course in sepsis and endotoxemia (129–131). A null mutation of G6PD causes chronic granulomatous disease (CGD)-like syndromes and is incompatible with life in hemizygous males (129, 130). Studies on severely injured African American male trauma patients indicated that the common African G6PD variants were associated with increased incidence of infection and sepsis, alterations in monocyte activation, and worsened secondary anemia as compared to individuals with the WT allele (133). The impact of G6PD polymorphic variants on female trauma patients has not been tested, but the X-linked distribution and mosaic expression in females could impact the trauma course contributing to sex-biased outcomes.

Another likely X-linked candidate gene is CYBB (gp91phox, NOX2) encoding for the catalytic subunits of NADPH oxidase, which is responsible for superoxide production by phagocytes during the oxidative burst. Mutations in this gene are the most frequent causes of CGD (134). CGD patients present recurrent bacterial and fungal infections due to inefficient killing of pathogens by phagocytes. Null mutation of this protein is usually fatal in hemizygous males due to recurrent infections and uncontrollable sepsis. Clinical symptoms are related to the type of mutations, which are rather heterogeneous and newly described mutations continue to emerge (135–138). Animal studies suggested that mice with WBC mosaicism for NOX2-WT and NOX2-KO have a dual advantage. In these mosaic animals, efficient bacterial killing was maintained whereas oxidative stress-mediated organ damage was diminished, thereby resulting in improved survival of mosaic mice as compared to homozygous WT or full NOX2 deficiency (50).

All the remaining immune-competent and apoptotic genes listed in Table 1 were reported to have dozens or some of them hundreds of exonic and intronic allelic variants as well as base changes in flanking regulatory regions. Reliable population frequency data are lacking for most of the genes listed and the associated clinical phenotypes are available only to those few mutations, which causes severe pathological phenotype. Nonetheless, conceptually, these polymorphic variants could contribute to increased X-linked cellular functional variability and immuno-modulation. Evidently, there could be many more X-linked polymorphisms impacting genes encoding for regulatory proteins, receptors, and enzymes of various metabolic functions, which may contribute either directly or indirectly to increased cellular variability *via* ChrX mosaicism and associated immuno-modulation.

Finally, it is worth mentioning that ChrX is one of the richest among chromosomes in encoding miRNAs many of which having immuno-regulatory functions (35, 139). Some of these miRNAs are polymorphic and were shown to impact T cell responses or their presence was associated with autoimmune conditions including Crohn’s disease and rheumatoid arthritis (35, 139, 140). Because miRNAs have multiple genetic targets within a cell, mosaic distribution of polymorphic miRNAs in females may also participate in producing unique X-linked phenotypes. Recent studies showed sex-biased roles of X-linked miRNAs in rheumatoid arthritis (140) consistent with this notion.

## Conclusion

We provided a set of facts, arguments, and supporting observations, which indicate that sex-related differences during the inflammatory response may be related to X-linked polymorphic genes and the associated increase in cellular variability in females. The fact that several key regulatory and metabolic proteins participating in signal transduction, apoptosis, carbohydrate metabolism, superoxide production and xenobiotic, and antioxidant defenses are encoded on the X chromosome and their polymorphic variants are widespread in the population strongly supports this hypothesis.

Inflammation is often considered to be a double-edged sword because it is required for the control and elimination of invading microorganisms; however, an overwhelming inflammatory response may lead to organ dysfunction or septic shock and death. In males, the cells of innate immunity carry one allelic compilation of the gene pool that may be adequate, hypo- or hyperresponsive under a pathological condition. In females, the two sets of innate immune cells differing in X-linked parental alleles with different regulatory and activation capacity could represent a more balanced and more adaptive system, which better adjusts to dynamically changing pathophysiology during the inflammatory response.

These differences in cellular functional variability between males and females and the male-biased X-linked inheritance pattern are likely contributors to sex hormone effects in causing sex-associated outcome differences in the population. Pursuing further this concept will be valuable because it can reveal novel aspects of cellular defense mechanisms, which could be utilized in developing improved therapeutic routines. Furthermore, testing for X-linked risk alleles together with the determination of ChrX skewing in females will help to stratify female subgroups or identify outliers within a cohort. The unique regulation of ChrX should be calculated into study designs and considered at the analytical stages of clinical studies investigating mixed cohorts especially with a focus on infection and immunity.

## Ethics Statement

This study was carried out in accordance with the recommendations of the Institutional Review Board of Rutgers-New Jersey Medical School with written informed consent from subjects in accordance with the Declaration of Helsinki. The protocol was approved by the Institutional Review Board of Rutgers-New Jersey Medical School.

## Author Contributions

ZS: primary contributor of developing the concept, planning and organizing experiments, analyzing data, and formulating the manuscript. GP, YQ, RD, and DL: participated in conceptual aspects, planning and analyzing experiments, and formulation of manuscript.

## Conflict of Interest Statement

The authors declare that the research was conducted in the absence of any commercial or financial relationships that could be construed as a potential conflict of interest.
